# Supplementary material for: Variations in elemental composition of rice (Oryza sativa L.) with different cultivation areas of Ethiopia
Source: PLoS One. 2023 Oct 19;18(10):e0290073. doi: 10.1371/journal.pone.0290073 (PMC10586638; doi:10.1371/journal.pone.0290073)
Supplement: Attachment — Submitted filename: Response to Reviewers.docx [file pone.0290073.s002.docx]

PONE-D-23-10177

Variations in Elemental Composition of Rice with Different Cultivation Areas of Ethiopia

Andrea Mastinu

Academic Editor, PLOS ONE

Journal Requirements:

When submitting your revision, we need you to address these additional requirements.

1. Please ensure that your manuscript meets PLOS ONE's style requirements, including those for file naming. The PLOS ONE style templates can be found at https://journals.plos.org/plosone/s/file?id=wjVg/PLOSOne_formatting_sample_main_body.pdf and 
<https://journals.plos.org/plosone/s/file?id=ba62/PLOSOne_formatting_sample_title_authors_affiliations.pdf>

**Author Response**: We thank the editor for handling our manuscript favorably. We have followed PLOS ONE’s style requirements.

2. In your Methods section, please provide additional information regarding the permits you obtained for the work. Please ensure you have included the full name of the authority that approved the field site access and, if no permits were required, a brief statement explaining why.

**Author Response**: Samples were collected from private farmlands, after permission was granted by the farmers to access their fields. This information is now provided in the method section.

3. We note that Figure (1) in your submission contain copyrighted images. All PLOS content is published under the Creative Commons Attribution License (CC BY 4.0), which means that the manuscript, images, and Supporting Information files will be freely available online, and any third party is permitted to access, download, copy, distribute, and use these materials in any way, even commercially, with proper attribution. For more information, see our copyright guidelines: http://journals.plos.org/plosone/s/licenses-and-copyright.
We require you to either (1) present written permission from the copyright holder to publish these figures specifically under the CC BY 4.0 license, or (2) remove the figures from your submission:

**Author Response**: The map is constructed from a freely available world shape file. This is now indicated in the figure caption.

(<https://datacatalog.worldbank.org/search/dataset/0038272/World-Bank-Official-Boundaries>)

1. You may seek permission from the original copyright holder of Figure (1) to publish the content specifically under the CC BY 4.0 license.

We recommend that you contact the original copyright holder with the Content Permission Form (http://journals.plos.org/plosone/s/file?id=7c09/content-permission-form.pdf) and the following text:
“I request permission for the open-access journal PLOS ONE to publish XXX under the Creative Commons Attribution License (CCAL) CC BY 4.0 (http://creativecommons.org/licenses/by/4.0/). Please be aware that this license allows unrestricted use and distribution, even commercially, by third parties. Please reply and provide explicit written permission to publish XXX under a CC BY license and complete the attached form.”
Please upload the completed Content Permission Form or other proof of granted permissions as an "Other" file with your submission.

In the figure caption of the copyrighted figure, please include the following text: “Reprinted from [ref] under a CC BY license, with permission from [name of publisher], original copyright [original copyright year].”

2. If you are unable to obtain permission from the original copyright holder to publish these figures under the CC BY 4.0 license or if the copyright holder’s requirements are incompatible with the CC BY 4.0 license, please either i) remove the figure or ii) supply a replacement figure that complies with the CC BY 4.0 license. Please check copyright information on all replacement figures and update the figure caption with source information. If applicable, please specify in the figure caption text when a figure is similar but not identical to the original image and is therefore for illustrative purposes only.

[Note: HTML markup is below. Please do not edit.]

Review Comments to the Author

Reviewer #1:

**Author Response**: We thank Reviewer #1 for reviewing our manuscript favorably.

The literature survey is not comprehensive. The following literature should be reviewed and cite in the Introduction:

Physical properties and chemical composition of three Ethiopian rice (Oryza sativa Linn.) varieties compared to tef [Eragrostis tef (Zucc.) Trotter] grain. <https://doi.org/10.51745/najfnr.3.6.180-185>

Effect of rice variety and location on nutritional composition, physicochemical, cooking and functional properties of newly released upland rice varieties in Ethiopia. Cogent Food And Agriculture 2021, 7(1):1945281. DOI: 10.1080/23311932.2021.1945281

Physicochemical, Nutritional Composition, Cooking, and Functional Properties of Newly Introduced Low Land Rice Varieties Grown in Ethiopia. Philippine Journal of Science 2020, 150(3):923-934. DOI: 10.56899/150.03.27

Global Geographical Variation in Elemental and Arsenic Species Concentration in Paddy Rice Grain Identifies a Close Association of Essential Elements Copper, Selenium and Molybdenum with Cadmium. Exposure and Health (2022). <https://doi.org/10.1007/s12403-022-00504-1>

**Author Response**: We have reviewed the suggested articles and the relevant information have been incorporated in the introduction section.

Table 1 should be deleted. The wavelength column should be incorporated in Table 5.

**Author Response**: Table 1 is deleted and wavelength column is incorporated in Table 5.

Comparison of levels of metals in this study with other studies reported from Ethiopia and other African countries should be given.

**Author Response**: We have compared the results of this study with other reported values from Ethiopia and some other countries in the results and discussion section.

References require carefully checking and editing.

Ref. [9], the article title should be corrected as in the other references.

Ref. [11], the article title should be corrected as in the other references.

Ref. [16], the article title should be corrected as in the other references.

Ref. [19], the article title should be corrected as in the other references.

Ref. [23], the article title should be corrected as in the other references.

Ref. [32], Bulletene should be Bulletin

**Author Response**: All the references have been carefully checked and edited.

Reviewer #2: Dear Authors

Your work is very interesting but still need some improvement to be suitable for publication.

**Author Response**: We thank Reviewer #2 for reviewing our manuscript favorably.

Please find attached file for comments and modifications.

**Author Response**: All the comments and modifications provided directly in the attached manuscript are incorporated.

The plant Latin name must added in the name of manuscript in full details (add author name) Oryza sativa L.

**Author Response**: The plant Latin name has been revised as *Oryza sativa* L.

Plant name along the manuscript must be in a short form as O. sativa.

**Author Response**: We have used the short form as *O*. *sativa*, after introducing the full name, along the manuscript.

Some editing errors must be corrected:

**Author Response**: We have thoroughly revised the manuscript according to the comments and suggestions provided in the attached manuscript.

Please determine the area of the studied areas.

**Author Response**: We have provided the names of the areas from which the samples were collected.

The family name of rice must be updated to Poaceae instead of Gramineae.

**Author Response**: We have updated the family name as Poaceae.
