# Supplementary material for: Variations in elemental composition of rice (Oryza sativa L.) with different cultivation areas of Ethiopia
Source: PLoS One. 2023 Oct 19;18(10):e0290073. doi: 10.1371/journal.pone.0290073 (PMC10586638; doi:10.1371/journal.pone.0290073)
Supplement: Attachment — Submitted filename: Response to Reviewers.docx [file pone.0290073.s004.docx]

PONE-D-23-10177R1

Variations in Elemental Composition of Rice (*Oryzasativa* L.) with Different Cultivation Areas of Ethiopia

Dear Andrea Mastinu

Academic Editor, PLOS ONE

Journal Requirements:

Please review your reference list to ensure that it is complete and correct. If you have cited papers that have been retracted, please include the rationale for doing so in the manuscript text, or remove these references and replace them with relevant current references. Any changes to the reference list should be mentioned in the rebuttal letter that accompanies your revised manuscript. If you need to cite a retracted article, indicate the article’s retracted status in the References list and also include a citation and full reference for the retraction notice.

**Author Response**: We thank the editor for handling our manuscript favorably. We have reviewed the reference list to ensure that it is complete and correct.

Reviewers' comments:

Reviewer #1: The manuscript has been revised appropriately according to my comments. The revised manuscript is acceptable for publication.

**Author Response**: We thank Reviewer #1 for reviewing our manuscript favorably.

Reviewer #2: The manuscript be better just few editing corrections are needed. Please follow attached file for corrections

**Author Response**: We thank Reviewer #2 for reviewing our manuscript favorably. We have incorporated all the comments and modifications provided directly in the attached manuscript.
